# *In Vitro* Antifungal Activity of *Burkholderia gladioli* pv. *agaricicola* against Some Phytopathogenic Fungi

**DOI:** 10.3390/ijms131216291

**Published:** 2012-12-03

**Authors:** Hazem S. Elshafie, Ippolito Camele, Rocco Racioppi, Laura Scrano, Nicola S. Iacobellis, Sabino A. Bufo

**Affiliations:** 1 Department of Science, University of Basilicata, Viale dell’Ateneo Lucano, 85100 Potenza, Italy; E-Mails: hazem.elshafie@yahoo.com (H.E.); rocco.racioppi@unibas.it (R.R.); laura.scrano@unibas.it (L.S.); sabino.bufo@unibas.it (S.A.B.); 2 School of Agricultural, Forestry, Food and Environmental Sciences, University of Basilicata, Viale dell’Ateneo Lucano, 85100 Potenza, Italy; E-Mail: nicola.iacobellis@unibas.it

**Keywords:** *Burkholderia gladioli* pv. *agaricicola*, antifungal activity, volatile organic compounds, extracellular enzymes, biological control

## Abstract

The trend to search novel microbial natural biocides has recently been increasing in order to avoid the environmental pollution from use of synthetic pesticides. Among these novel natural biocides are the bioactive secondary metabolites of *Burkholderia gladioli* pv. *agaricicola* (*Bga*). The aim of this study is to determine antifungal activity of *Bga* strains against some phytopathogenic fungi. The fungicidal tests were carried out using cultures and cell-free culture filtrates against *Botrytis cinerea*, *Aspergillus flavus*, *Aspergillus niger*, *Penicillium digitatum*, *Penicillium expansum*, *Sclerotinia sclerotiorum and Phytophthora cactorum.* Results demonstrated that all tested strains exert antifungal activity against all studied fungi by producing diffusible metabolites which are correlated with their ability to produce extracellular hydrolytic enzymes. All strains significantly reduced the growth of studied fungi and the bacterial cells were more bioactive than bacterial filtrates. All tested *Bulkholderia* strains produced volatile organic compounds (VOCs), which inhibited the fungal growth and reduced the growth rate of *Fusarium oxysporum* and *Rhizoctonia solani*. GC/MS analysis of VOCs emitted by strain *Bga* 11096 indicated the presence of a compound that was identified as 1-methyl-4-(1-methylethenyl)-cyclohexene, a liquid hydrocarbon classified as cyclic terpene. This compound could be responsible for the antifungal activity, which is also in agreement with the work of other authors.

## 1. Introduction

The biological control of plant diseases, insects and nematodes by microorganisms has been proposed as an alternative or a supplement to chemical control [1,2]. The biological control activity of several bacteria is exerted either directly through antagonism of pathogen development, or indirectly by eliciting a plant-mediated resistance response [3]. Mechanisms responsible for antagonistic activity include: inhibition of the pathogen growth; competition for colonization sites, nutrients and minerals; parasitism; and, mycophagy [4–6]. It is a common strategy of bacterial antagonists to inhibit the plant pathogens by excretion of antimicrobial metabolites (AMMs). Well-known AMMs are antibiotics, toxins and bio-surfactants [7]. Recently, it was demonstrated that volatile organic compounds (VOCs), including hydrocarbons, alcohols, ketones, aldehydes, ethers, esters, terpenes, terpene derivatives, and several heteroaromatic compounds produced by some bacteria can influence the growth of fungi [8–10].

*Burkholderia gladioli* pv. *agaricicola* (*Bga*) is an important pathogen in the mushroom industry, since it causes soft rot of *Agaricus bitorquis* and *A. bisporus*[11–13]. *Burkholderia gladioli* possesses a great potential as a plant pathogen antagonist [14–17]. In particular, the LMG 18920 strain of *B. gladioli* (*Pseudomonas antimicrobica*) showed antagonistic activity *in vitro* against a wide range of fungal and bacterial species [15–17]. Metabolites produced by this bacterium caused a significant inhibition of the conidial germination of *Botrytis cinerea*[16,17]. Recent experiments performed in an apricot tree field proved its efficacy as a biocontrol agent against *Monilia laxa*[18], and it also showed completely *in vitro* inhibition for the conidial germination of *Penicillium digitatum* and *Penicillium expansum*[18]. Components of *B. gladioli* showed high ability to produce extracellular hydrolytic enzymes such as chitinase, protease, cellulase, amylase and glucanase that may have direct effect on the fungal growth [19–23]. Among these enzymes, chitinase is able to degrade chitin in chitooligosaccharides which have numerous and important applications in pharmaceutical and industrial fields [21]. Jijakli and Lepoivre [22] demonstrated that *in vivo* and *in vitro* antifungal activity of *Pichia anomala* are directly related to a β-1,3-exoglucanase enzyme production. Saligkarias *et al.*[23] reported that protease enzyme has been shown to have antifungal activity against phytopathogenic fungi *in vivo* and *in vitro*.

This work aims to evaluate the bioactivity of of four *Bga* strains and their culture filtrates against the plant pathogenic and plant-associated fungi: *B. cinerea* Pers., *Aspergillus flavus* Link ex Gray*, Aspergillus niger* van Tieghem, *P. digitatum* Sacc., *P. expansum* Link, *Sclerotinia sclerotiorum* (Lib.) de Bary *and Phytophthora cactorum* (Leb. and Cohn) Schröeter, and to assess the effect of *Bga*-produced VOCs on the growth rate and suppression of *Fusarium oxysporum* Schlect and *Rhizoctonia solani* J.G. Kühn.

## 2. Results and Discussion

### 2.1. Screening of Antifungal Activity

Many species of bacteria have been reported to be able to suppress the growth of several fungal and bacterial pathogens and nematodes through antagonist behavior. Among them are included: *Agrobacterium radiobacter*, *Bacillus subtillis*, *B. megaterium*, *B. pumilus*, *B. cereus*, *Burkholderia cepacia*, *Pasteuria penetrans*, *Pseudomonas aeruginosa*, *P. fluorescent*, *P. putida*, *P. syringae*, *Serratia marcescens*, *Serratia sp*., *and Sternotrophomonas maltophilia*[24–28].

In this study, a preliminary experiment was carried out to evaluate in the dual plate assay the ability of four *B.g.* pv. *agaricicola* strains and of their respective culture filtrates to inhibit the growth of the phytopathogenic and plant-associated fungi *B. cinerea*, *A. flavus*, *A. niger*, *P. digitatum*, *P. expansum*, *S. sclerotiorum* and *P. cactorum*. Results showed that *Bga* strains and their culture filtrates inhibited the growth of the selected fungi. Similar antifungal activities have been reported for *Burkholderia* spp. [19,29,30]. Although in some of the cases the microbial activity was proposed to be mostly due to the fungal cell wall lytic enzymes [30], other antifungal metabolites such as antibiotics, Fe-chelating siderophores, ammonia and cyanide were also reported [19].

Mycelia growth of target fungi in dual plate assays was significantly different from the control (*p* < 0.05). Strain ICMP 11096 of *B.g.* pv. *agaricicola* revealed the highest significant fungitoxicity against *A. flavus*, *A. niger*, *B. cinerea*, *P. expansum*, *P. cactorum* and *S. sclerotiorum* (Table 1). In contrast, strain *Bga* ICMP 12220 showed the highest significant antifungal activity against *P. digitatum* (Table 1).

### 2.2. Comparing the Fungitoxicity of Bacterial Culture and Filtrate

**Figure 1:**
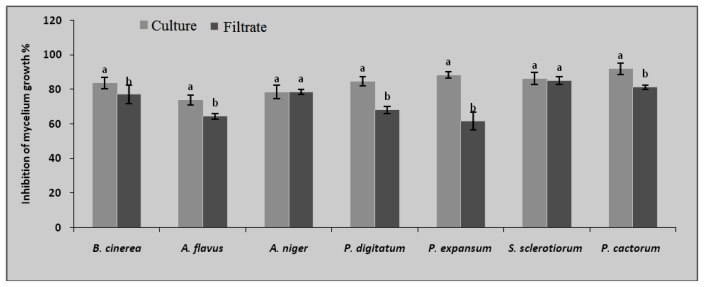
Percentage of growth inhibition (PGI) for cell-free culture filtrate and bacterial culture of *Bga* ICMP 11096. Bars with different letters indicate mean values significantly different at *p* < 0.05 according to Tukey B test. Data are expressed as mean of three replicates ± SD.

*Bga* strain ICMP 11096 has been selected for comparing the fungitoxicity of either whole culture or cell-free culture filtrate according to the percentage of fungal mycelium inhibition (PGI). The culture filtrate of bacterial strain ICMP 11096 inhibited the growth of all tested fungi on PDA agar plate assay (Figure 1). The size of the inhibition zones produced by bacterial cells was significantly higher than that produced by the corresponding culture filtrate. On the other hand, there is no significant difference between filtrate and bacterial cells regarding *A. niger* and *S. sclerotiorum*, which achieved the same fungitoxicity percentage.

### 2.3. Extracellular Hydrolytic Activities

*Bga* strains showed chitinase, protease and glucanase activities, forming clear zones on chitin, skim milk and Lichenan media plates ranging from 13 to 25 mm, 25 to 36 mm and 20 to 26 mm, respectively. On the contrary, the selected strains did not show cellulase, amylase, pectinase and polygalacturonase activities. The production of chitinase and glucanase could contribute to the growth inhibition control of the phytopathogenic fungi through degradation of fungal cell walls [31]. Protease enzymes as cell wall lytic enzymes, may play a significant role in degrading the fungal cell wall, since fungal cell wall “skeletal” components are embedded in a protein matrix [23] and proteolytic activity is necessary for the lysis of whole fungal cells.

### 2.4. Antifungal Activity of Volatile Organic Compounds

**Figure 2:**
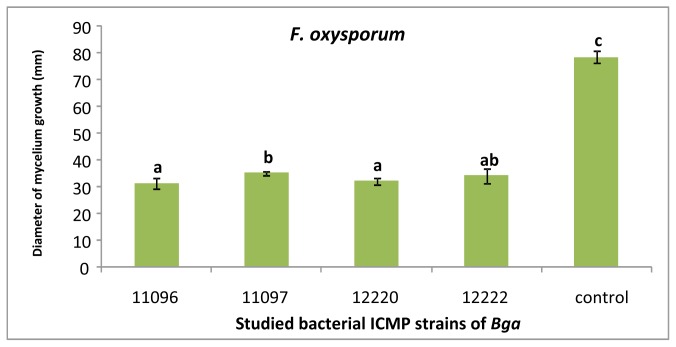
Antifungal activity of volatile organic compounds of *Bga* strains *vs. F. oxysporum* (5 days growth). Bars with different letters indicate mean values significantly different at *p* < 0.05 according to Duncan test. Data are expressed as mean of three replicates ± SD.

**Figure 3:**
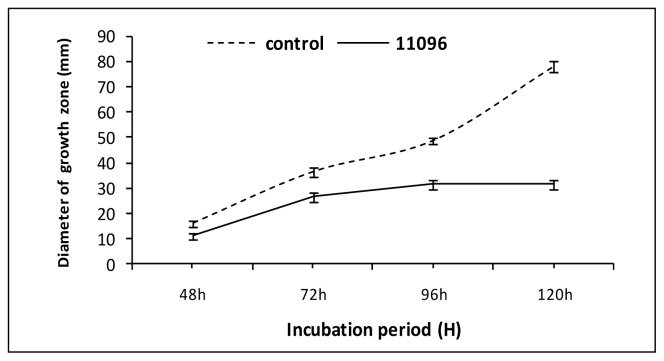
Effect of volatile secondary metabolites of *Bga* ICMP11096 on the growth rate of *F. oxysporum.* Data are expressed as a mean of three replicates ± SD.

**Figure 4:**
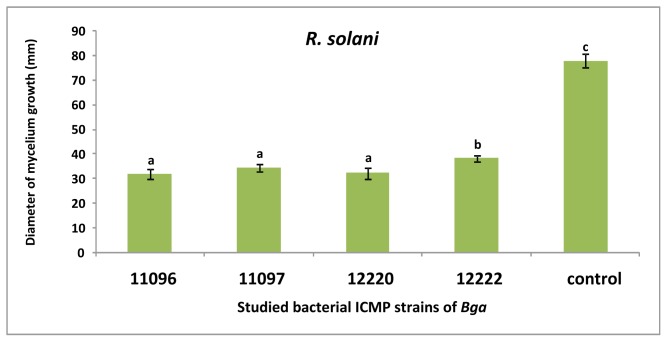
Antifungal activity of volatile secondary metabolites of *Bga* strains *vs. R. solani* (five days). Bars with different letters indicate mean values significantly different at *p* < 0.05 according to Duncan test. Data are expressed as mean of three replicates ± SD.

Strains of *Bga* produced VOCs which reduced the mycelium growth of *F. oxysporum* (Figure 2). Strains *Bga* ICMP11096 and ICMP12220 showed the highest significant reduction of fungal growth compared to other studied strains. Strain *Bga* ICMP11096 has been selected for studying the effect of its VOCs on the fungal growth rate of *F. oxysporum* (Figure 3). After four days of incubation, the fungal growth appeared to be almost stopped. Similar results were obtained when the above assays were performed versus *R. solani* (Figure 4). Strains *Bga* ICMP11096, ICMP11097 and ICMP 12220 showed the highest significant reduction of fungal growth compared to strain Bga ICMP 12322. In contrast, Kai *et al.*[32] found that volatiles of *Pseudomonas* spp., *Serratia* spp., *Stenotrophomonas* spp. drastically inhibited the growth of *R. solani*, while a moderate or no inhibition was observed with the volatile substances of *B. cepacia and Staphylococcus epidermidis*, respectively.

**Figure 5:**
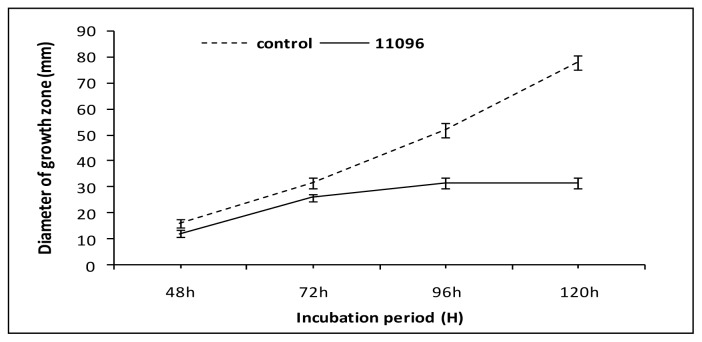
Effect of volatile secondary metabolites of *Bga* 11096 on growth rate of *R. solani.* Data are expressed as mean of three replicates ± SD.

Also in this case the strain *Bga* ICMP 11096 was able to block completely the mycelium growth after four days of incubation (Figure 5).

### 2.5. Analysis of VOCs by GC-MS

**Figure 6:**
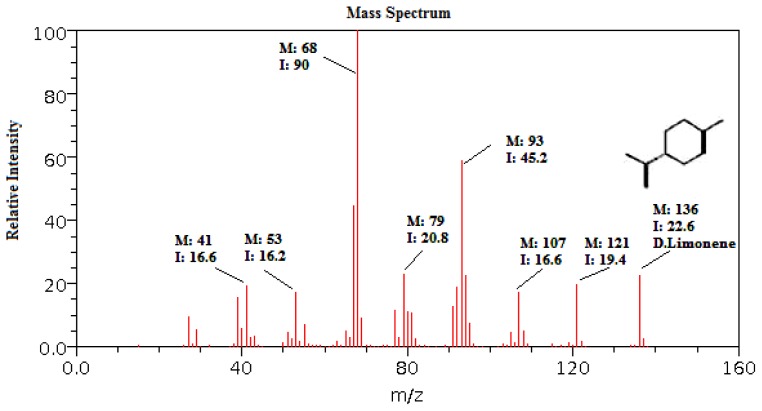
Mass spectrum of the main volatile compound produced by *Bga* ICMP11096, a cyclic terpene (an isomer of limonene).

GC-MS analysis of VOCs produced by *Bga* strain ICMP 11096 indicated the presence of 1-methyl-4-(1-methylethenyl)-cyclohexene, which was detected at retention time 11.61 min and has a molecular weight of 136 (Figure 6). This isolated main volatile compound is a liquid hydrocarbon that can be classified as cyclic terpene. This compound could be responsible for the antifungal activity of *Bga* strain ICMP 11096 against all studied phytopathogenic fungi.

## 3. Experimental Section

### 3.1. Bacterial and Fungal Strains Used in this Study

The phytopathogenic as well as plant-associated fungi *B. cinerea*, *A. flavus*, *A. niger*, *P. digitatum*, *P. expansum*, *S. sclerotiorum*, *P. cactorum*, *F. oxysporum* and *R. solani*, were grown and maintained on potato dextrose agar (PDA) or corn meal agar (CMA). *Bga* strains ICMP 11096, 11097, 12220 and 12322, isolated from *Agaricus bitorquis* (Quelet) Saccardo, were obtained from International Collection of Micro-organisms from Plants (ICMP) (Landcare Research, Auckland, New Zealand). Bacterial strains were maintained as lyophils at 4 °C and subcultures were obtained on the medium King Agar B (KB) for 48 h at 25 °C.

### 3.2. Antifungal Assays

The antifungal activity of of *Bga* strains against fungi was carried out by growing the bacterial strains on KB medium and incubated at 30 °C for 24 h. Then 1 cm^2^ agar disc was cut off with a cork borer and placed onto the surface of fungal plates containing PDA medium using bacterial disc method [33]. The plates were incubated at 25 °C for 4–5 days.

The antifungal activity of cell-free filtrates of *Bga* against fungi grown on PDA was conducted using the diffusion method [34]. Erlenmeyer flask containing 150 mL of liquid minimal mineral medium (MM) were inoculated with 1.5 mL bacterial suspension containing 10^8^ CFU mL^−1^ and incubated under rotary shaking (180 rpm) at 25 °C for 7 days [35]. Then, the culture was centrifuged at 20,000× *g* for 15 min and membrane filtered (Millipore, 0.20 μm), then 10 μL of filtrate was deposited on 14 mL solid PDA medium inoculated with 1 cm^2^ of fungal disc. After 4–5 days of incubation, the diameter of the fungal colonies were scored and measured in mm.

### 3.3. Comparing the Fungitoxicity of Bacterial Cultures and Filtrates

According to the screening test, the agar diffusion method was used for testing the antifungal activity of cell-free culture filtrates for the highest bioactive antagonistic bacterial strain of *Bga*. The antifungal activity of bacterial cultures and filtrates was determined against all studied phytopathogenic fungi and the fungitoxicity was expressed as percentage of growth inhibition (PGI) and calculated according to Zygadlo et al. formula [36] (Equation 1).

(1) PGI (%)=100 (GC-GT)/GC

where GC represents the average diameter of fungi grown in PDA (control); GT represents the average diameter of fungi cultivated on the treated PDA dish containing the antagonistic bacteria.

### 3.4. Extracellular Hydrolytic Activities

Chitinase and protease activities of *Bga* strains were determined according to Tahtamouni *et al.*[37] on plates of KB containing chitin (1%) and skim milk (1%) respectively. Cellulase activity was detected according to the Essghaier *et al.* method [38] using carboxymethyl cellulose (0.4%). Glucanase activity was detected according to Teather and Wood method [39] using Lichenan (0.2%).

Amylase, pectinase and polygalacturanase activities were detected using soluble starch (1%), pectin (0.5%) and polygalacturanic acid (1%), respectively [40,41]. After incubation at 30 °C for 4–5 days, the plates were flooded with specific staining solutions; congo red 0.03% for chitinase, cellulase and glucanase; lugol solution for amylase; CTAB 2% for pectinase; and, ruthenium red 0.1% for polygalacturanase. The enzymatic activity was taken as evidence by appearance of clear zones around the colonies and their diameters were measured in millimeters.

### 3.5. Antifungal Activity of Volatile Secondary Metabolites

The assays were carried out by plating 100 μL of bacterial suspension containing 10^8^ CFU mL^−1^ on the surface of Petri dishes containing 14 mL KB incubated at 25 °C for 24 h, whereas fungi were grown on Petri dishes containing 14 mL PDA incubated at 30 °C for 96 h. The bottom parts of the plates were tightly joined with parafilm and incubated in the darkness at 30 °C for 4–5 days. Fungitoxicity of volatile metabolites was expressed by measuring the diameter of mycelium growth (mm). All treatments of the experiment were carried out twice with three replicates. The highest bioactive antagonistic strain was selected to study the effect of its volatile metabolites on fungal growth rate using the Evmert *et al.* method [42].

### 3.6. Analysis of Volatile Substances by GC-MS

For the production of VOCs, the *Bga* strains were grown on KB and 100 μL bacterial suspensions of about 10^8^ CFU mL^−1^ were used to inoculate 25 mL glass vials containing 10 mL KB. Vials were sealed with a plastic cap fitted with a Teflon/silicon septum and then incubated for 5 days at 25 °C.

An SPME fiber coated with 100 μm of nongrafted poly-dimethylsiloxane (PDMS) phase (Supelco 57300-U on a Supelco 57330 support) was conditioned for 1 h at 250 °C in helium stream; after that, the fiber was inserted into the headspace of the glass vial containing the inoculated strain and was maintained there for 20 min at 25 °C. The fiber was then introduced into the injection port of a HP 6890 plus gas chromatograph equipped with a Phenomenex Zebron ZB-5 MS capillary column (30 m × 0.25 mm ID × 0.25 μm film thickness). The HP5973 mass selective detector was used (mass range: 15–800 mAU; scan rate: 1.9 scan/s; EM voltage: 1435), helium at 0.8 mL min^−1^ was adopted as carrier gas. The injection port, equipped with glass insert (internal diameter 0.75 mm) was splitless at 250 °C. The desorption time of 1.0 min was used. The oven temperature was fixed at 40 °C for 2 min, then increased up to 250 °C (8 °C min^−1^); finally, it was maintained for 10 min. All the analyses were performed in triplicate. A blank run was performed after each analysis in order to confirm that no residual compound was polluting the fiber or the column. The chromatograms obtained from the total ion current (TIC) were integrated without any correction for coelutions and results were expressed as percent of the total area of peaks. All the peaks were identified from their mass spectra by comparison with spectra in Wiley 6N and NIST98 libraries.

### 3.7. Statistical Analysis

The experimental data of the antifungal activity of diffusible and volatile bioactive substances of *Bga* strains on the phytopathogenic and plant-associated fungi growth were analyzed statistically by one-way ANOVA, Duncan and Tukey B *post hoc* test using SPSS statistical software (version 13.0, Prentice Hall: Chicago, IL, USA, 2004).

## 4. Conclusions

The genus *Burkholderia* in recent years has been phylogenetically well defined, consisting of species that are functionally remarkably diverse [43]. In fact, *Burkholderia* species have been isolated from many different environmental niches, including soil and water, and can form associations with plants, animals and humans. Several *Burkholderia* spp. are widespread in nature and some of them are considered to be beneficial in the natural environment [15]. *Burkholderia gladioli* pv. *agaricicola* is an important pathogen in the mushroom industry; it causes soft rot of *Agaricus bitorquis* and *A. bisporus*[11–13].

In the present study we verified the biological and metabolic properties of selected strains of *Bga*, which can be exploited for the biological control of fungal diseases. Strains of *Bga* have been shown to antagonize a wide range of important phytopathogenic and plant-associated fungi, including *B. cinerea*, *A. flavus*, *A. niger*, *P. digitatum*, *P. expansum*, *S. sclerotiorum* and *P. cactorum.* In other studies the ability of this mushroom pathogen to inhibit the growth of yeast, Gram-positive and Gram-negative bacteria has been reported [11,12,18–20]. The highest level of antifungal activity was recorded when bacterial cultures of *Bga* were used in antagonist dual tests. This antifungal activity could be attributed to the ability of studied bacterial strains to produce some extracellular hydrolytic enzymes such as chitinase, protease and glucanase. The production of hydrolytic enzymes is considered one of the most important mechanisms explaining the biocontrol of phytopathogenic fungi by the degradation of fungal cell walls [44].

On the other hand, *Bga* produced VOCs, which apparently reduced the fungal growth rate of *F. oxysporum* and *R. solani* and completely blocked their growth after five days of incubation. GC-MS analysis of bulk VOCs produced by *Bga* strain ICMP11096 showed that the main compound was 1-methyl-4-(1-methylethenyl)-cyclohexene, a cyclic terpene considered one of the most common D isomer of limonene. This VOC is apparently responsible for the antifungal activity against phytopathogenic and plant-associated fungi in agreement with Ayoola *et al.*[45] that reported the antimicrobial activity of D limonene against a wide range of organisms including fungi. On the other hand, Hossain *et al.*[46] showed the remarkable antifungal effect of *Orthosiphon stamineus* essential oil against some phytopathogenic fungi. The activity of *O. stamineus* essential oil has been attributed to the presence of caryophyllene, humulene, elemene, bourbonene, pinene, caryophyllene oxide, camphene and limonene, which significantly inhibited the growth of all the phytopathogens tested as *B. cinerea*, *R. solani*, *Fusarium solani*, *Colletotricum capsici* and *Phytophthora capsici.*

We can speculate that the potential biological control of selected *Bga* strains could be correlated with their ability to produce extracellular hydrolytic enzymes and diffusible and volatile secondary metabolites. Although the preliminary results of this study suggest *Bga* as biocontrol agents for the diseases caused by tested fungi, further investigation appears necessary in this regard, as well as the chemical and biological characterization of bioactive secondary metabolites, which are currently under examination in our laboratories.

## Figures and Tables

**Table 1:** *In vitro* antagonistic activity of bacterial cultures and cell-free culture filtrates recorded as the mean diameter of fungal mycelium growth (mm) ± Standard Deviation. Values followed by the same letter in each vertical column are not significantly different according to Duncan test at *p* < 0.05. Data were obtained on three replicates.

Fungal pathogens (Mean diameter of fungal mycelium growth mm ± SD)							

Bacterial Filtrates	*B. cinerea*	*A. flavus*	*A. niger*	*P. digitatum*	*P. expansum*	*S. sclerotiorum*	*P. cactorum*
11096	10.5 ± 2.12 c	13.0 ± 1.41 c	11.0 ± 1.41 c	11.5 ± 2.12 b	11.0 ± 1.41 b	6.5 ± 0.71 c	7.5 ± 0.71 b
11097	15.5 ± 2.12 b	18.0 ± 1.41 b	15.0 ± 1.41 b,c	13.0 ± 1.41 b	13.5 ± 0.71 b	9.5 ± 0.71 b	10.0 ± 2.83 b
12220	11.0 ± 1.41 b,c	16.5 ± 0.71 b,c	16.0 ± 1.41 b	9.5 ± 0.71 b	14.5 ± 0.71 b	8.5 ± 0.71 b,c	11.0 ± 1.41 b
12322	14.0 ± 1.41 b,c	16.5 ± 2.12 b,c	15.0 ± 1.41 b,c	7.5 ± 0.71 b	13.5 ± 2.12 b	9.5 ± 0.71 b	8.5 ± 0.71 b
Control	46.0 ± 1.41 a	36.5 ± 2.12 a	47.0 ± 2.83 a	36.0 ± 4.24 a	27.0 ± 2.83 a	44.0 ± 1.41 a	40.0 ± 1.41 a
**Bacterial Cultures**	***B. cinerea***	***A. flavus***	***A. niger***	***P. digitatum***	***P. expansum***	***S. sclerotiorum***	***P. cactorum***
11096	5.5 ± 1.4 c	12.6 ± 0.7 c	9.0 ± 2.1 c	6.0 ± 1.4 c	5.0 ± 2.1 c	6.0 ± 1.4 c	2.5 ± 0.7 c
11097	8.5 ± 2.1 b	17.0 ± 0.7 b	14.0 ± 2.1 b	8.5 ± 2.8 b	8.0 ± 2.8 b	11.5 ± 2.5 b	5.5 ± 0.7
12220	9.0 ± 1.4 b	14.0 ± 1.4 b,c	13.0 ± 2.1 b	4.5 ± 0.7 b,c	6.0 ± 2.1 b,c	9.5 ± 2.1 b	3.5 ± 0.7 b,c
12322	8.0 ± 2.1 b	19.0 ± 1.4 b	14.0 ± 1.4 b	11.5 ± 2.1 b	9.5 ± 2.1 b	9.5 ± 0.7 b	7.5 ± 0.7
Control	27.5 ± 2.1 a	48.0 ± 2.8 a	42.0 ± 2.1 a	39.0 ± 1.4 a	47.0 ± 2.8 a	44.5 ± 2.1 a	31.5 ± 2.1 a
